# Development and clinical validation of a one-step pentaplex real-time reverse transcription PCR assay for detection of hepatitis virus B, C, E, Treponema pallidum, and a human housekeeping gene

**DOI:** 10.1186/s12879-023-08240-w

**Published:** 2023-05-25

**Authors:** Miaomiao Li, Yan Lv, Dawei Cui, Yushan Xu, Mengjiao Lin, Xiaojun Zhang, Yongjun Wang, Cuifen Shen, Jue Xie

**Affiliations:** 1 Department of Blood Transfusion, The First Affiliated Hospital, Zhejiang University School of Medicine, Hangzhou, 310003 China; 2 Ningbo Central Blood Station, Ningbo, 315099 China; 3 Key Laboratory of Blood Safety Research of Zhejiang Province, Zhejiang Province Blood Center, Hangzhou, 310052 China; 4 Department of Clinical Laboratory, Huzhou Central Hospital, Huzhou, 313000 China

**Keywords:** Blood safety, Hepatitis B Virus, Hepatitis C Virus, Hepatitis E Virus, Treponema pallidum, Pentaplex real-time RT-PCR, Serology

## Abstract

**Background:**

With the safety of blood transfusion being a major public health concern, the development of a rapid, sensitive, specific, and cost-effective multiplex PCR assay for simultaneous detection of hepatitis B virus(HBV), hepatitis C virus (HCV), hepatitis E virus (HEV), and Treponema pallidum(T. pallidum) in blood is crucial.

**Methods:**

Five primer pairs and probes were designed towards conserved regions of target genes and used to establish a one-step pentaplex real-time reverse transcription PCR(qRT-PCR) assay for simultaneous detection of HBV, HCV, HEV, T. pallidum, and RNase P(housekeeping gene), providing sample quality check. The clinical performance of the assay was further determined with 2400 blood samples from blood donors and patients in Zhejiang province, and compared the results with commercial singleplex qPCR and serological assays.

**Results:**

The 95% limit of detection(LOD) of HBV, HCV, HEV, and T. pallidum were 7.11 copies/µL, 7.65 copies/µL, 8.45 copies/µL, and 9.06 copies/µL, respectively. Moreover, the assay has good specificity and precision. Compared to the singleplex qPCR assay, the novel assay for detecting HBV, HCV, HEV, and T. pallidum presented 100% clinical sensitivity, specificity, and consistency. Several discrepant results between serological and pentaplex qRT-PCR assays were found. Of 2400 blood samples, there were 2(0.08%) HBsAg positive samples, 3(0.13%) anti-HCV positive samples, 29(1.21%) IgM anti-HEV positive samples and 6(0.25%) anti-T. pallidum positive samples proven negative in nucleic acid detection. 1(0.04%) HBV DNA positive sample and 1(0.04%) HEV RNA positive sample were detected negative by serological testing.

**Conclusions:**

The developed pentaplex qRT-PCR is the first assay on simultaneous, sensitive, specific, and reproducible detection of HBV, HCV, HEV, T. pallidum, and RNase P in a single tube. It could detect pathogens in blood during the window period of infection and is a good tool for effectively screening blood donors and early clinical diagnosis.

## Introduction

Blood transfusion saves lives and improves health, but it can also be a vector for harmful infectious diseases, such as hepatitis and syphilis. Hepatitis B virus(HBV), hepatitis C virus (HCV), and Treponema pallidum(T. pallidum) are common transfusion-transmitted pathogens that pose a major threat to blood safety [1]. The positive rates of HBV, HCV, and T. pallidum infections in blood donation worldwide were 0.03–3.70%,0.02–1.03%, and 0.05–0.90%, respectively [2]. Screening blood donations for transfusion-transmitted infections(TTIs) is one of the most important measures for blood transfusion safety. The World Health Organization (WHO) recommends that all donated blood should be strictly tested for HBV, HCV, and T. pallidum according to quality system requirements [3].

Since the 1970s, global implementation of serological assays for HBV surface antigen (HBsAg) and antibodies to HCV(anti-HCV) in blood donors has significantly reduced the risk of transfusion-transmitted HBV and HCV. However, achieving “zero risk” is still a huge challenge because there is still the possibility of HBV and HCV transmission from seronegative donors within the window period of infection [4, 5]. Some studies have reported the effectiveness of nucleic acid testing(NAT) in reducing the residual risk of TTIs during the window period of HBV and HCV infections [6–10].

After the serological screening of blood donors in the 1940s, the number of cases of syphilis transmitted by blood transfusion has distinctly reduced [11]. However, the serological assay has inherent limitations: diminished sensitivity in the first and late stages of syphilis and the risk of false positive reactions [12]. Antibodies to T. pallidum(anti-T. pallidum) are generally undetectable during the first 2–6 weeks of infection when the infectivity of donors is considered to be the highest [13]. The PCR assay for detecting T. pallidum DNA has been verified as having potential in syphilis diagnosis, and it can improve syphilis diagnosis, especially in seronegative people [14, 15].

Hepatitis E virus (HEV) infection leads to acute viral hepatitis, usually asymptomatic and self-limiting in immunocompetent people. While HEV infection tends to develop into chronic infection, which can advance to cirrhosis and even liver failure in immunocompromised people, tumor patients, pregnant women, and liver disease patients with pre-existing HBV or HCV infections, meanwhile overall mortality has increased [16]. HEV has emerged as a TTI and constituted a new threat to blood transfusion safety, with a first case of transfusion-transmitted hepatitis E formally reported in 2002 in Hokkaido, Japan [17]. The necessity for mandatory HEV screening among blood donors remains controversial [18]. Several developed countries have employed blood screening strategy for HEV [19]. While many HEV endemic countries and areas, such as China, do not conduct routine HEV infection screening for blood donors [20]. Between 0.0013% and 0.281% of asymptomatic blood donors suffer from HEV viremia worldwide [21]. However, a recent meta-analysis concluded that only 26.6% of viremic blood units were positive for anti-HEV IgM and IgG [22]. Serological testing alone may not offer effective HEV screening for blood donors. The screening for HEV RNA in blood donations remains the only effective measure to prevent transfusion-transmitted HEV so far [19]. Furthermore, HEV RNA testing is necessary to diagnose or exclude HEV infection in immunocompromised hosts [23].

Along with molecular technology development, real-time PCR(qPCR) has become the preferred tool for laboratory diagnosis of pathogen infections. Multiplex qPCR can detect multiple targets simultaneously in one tube, which has the advantages of high throughput, fast testing speed, convenient procedure, low contamination risk, and low testing cost [24, 25]. A variety of singleplex and multiplex qPCR assays have been developed for detecting HBV, HCV, HEV, and T. pallidum over the last few decades [14, 26, 27]. Nevertheless, a pentaplex qRT-PCR assay for simultaneously testing these four pathogens has yet to be reported so far.

In the present study, we have developed a pentaplex qRT-PCR assay with Taqman probes allowing simultaneous, sensitive, specific, and reproducible detection of HBV, HCV, HEV, T. pallidum, and a human housekeeping control gene(RNase P) related nucleic acids in blood. The novel assay provides an optional blood screening strategy for blood stations that have implemented or plan to implement HEV screening for blood donors in the future.

## Methods

### Blood samples collection and nucleic acids extraction

The positive blood samples of hepatitis A virus(HAV), Epstein-Barr virus (EBV), herpes simplex virus(HSV), cytomegalovirus (CMV), and human immunodeficiency virus (HIV) confirmed by PCR were obtained from The First Affiliated Hospital, Zhejiang University School of Medicine, and used to detect the specificity of the pentaplex qRT-PCR. To evaluate the clinical performance of the pentaplex qRT-PCR assay, we collected 1200 blood samples from blood donors at the Ningbo Central Blood Station and Zhejiang Province Blood Center, and 1200 blood samples from inpatients of Huzhou Central Hospital and The First Affiliated Hospital, Zhejiang University School of Medicine from March 2021 to March 2022. In this research, all participants signed the written informed agreement. This study was conducted with the approval of the Ethics Committee of The First Affiliated Hospital, Zhejiang University School of Medicine(Reference Number2020-856).

According to the manufacturer’s instructions, nucleic acids were extracted from 200 µL of blood sample with the DNA/RNA Extraction Kit II (Geneaid Biotech, Taiwan, China). The extracts were dissolved with 50 µL RNase-free water and stored at − 80 °C before use.

### Design of primers and probes

DNASTAR software (7.1 version) was used for multiple sequence alignment to select the highly conserved surface(S) region of HBV, the 5’ untranslated region(5’ UTR) of HCV, open reading frame-2 (ORF2) region of HEV, and T. pallidum 47 kDa lipoprotein(TPP47) region of T. pallidum as the target genes, respectively, based on whole genome sequences of HBV, HCV, HEV, and T. pallidum in GenBank. Primers and probes for these target gene sequences were designed with Primer Premier 5.0 software. Additionally, primers and a probe for specific detection of RNase P were designed towards the ribonuclease P protein subunit p30(RPP30) region. The specificity of all primers and probes was checked by the BLAST (https://blast.ncbi.nlm.nih.gov/Blast.cgi). No significant similarities were observed. All primers and probes were synthesized by Shanghai Sangon Biotechnology Co., Ltd(Shanghai, China) and were shown in Table 1.

**Table 1:** Primers and probes for detecting HBV, HCV, HEV, T. pallidum, and RNase P

Species	Gene target	Primer/probe	Primer/probe sequence	Length (bp)	Product size(bp)	Concentration((µM)
HBV	S	Forward	5′-TGTGTCTGCGGCGTTTTATC-3′	20	139	0.2
	S	Reverse	5′-CATGGTCCCGTGCTGGTAG-3′	19		0.2
	S	Probe	5′-VIC-ATCCTGCTGCTATGCCTCATCTTCTTG-BHQ1-3′	27		0.2
HCV	5’ UTR	Forward	5′-TAGCCGAGTAGTGTTGGGTC-3′	20	95	0.1
	5’ UTR	Reverse	5′-TCATGGTGCACGGTCTACG-3′	19		0.1
	5’ UTR	Probe	5′-CY5-TACTGCCTGATAGGGTGCTTGCG-BHQ3-3′	23		0.1
HEV	ORF2	Forward	5′-ATCTTGCTGACACGCTTCTCG-3′	21	158	0.4
	ORF2	Reverse	5′-AGCTATACCCTTATCCTGCTGA-3′	22		0.4
	ORF2	Probe	5′-TAMRA-GTCTCAGCCAATGGCGAGCCGAC-BHQ2-3′	23		0.4
T.pallidum	TPP47	Forward	5′-AGGTAAGCAGCATGGAGAGC-3′	20	117	0.1
	TPP47	Reverse	5′-AGCCATCAGCCCTTTTCAGC-3′	20		0.1
	TPP47	Probe	5′-FAM-CCGCACGACCTTGTGGTAGACACG-BHQ1-3′	24		0.1
RNase P	RPP30	Forward	5′-GGCGGTGTTTGCAGATTT-3′	18	76	0.1
	RPP30	Reverse	5′-AGCGGCTGTCTCCACAAG-3′	18		0.1
	RPP30	Probe	5′-ROX-GGGTTCTGACCTGAAGGCTCTGC-BHQ2-3′	23		0.1

### Plasmid standards synthesis

The S region of HBV, 5’ UTR of HCV, ORF2 region of HEV, TPP47 region of T. pallidum, and RPP30 region of RNase P were synthesized and cloned into pUC57 Vector. The cloned strain of pUC57 was cultured in Escherichia coli DH5α, and then plasmid was extracted. The plasmid concentration was measured by NanoDrop (Thermo Fisher Scientific, USA) and converted into copy number according to the formula(plasmid copy number/µL= [6.02 × 10^23^ × plasmid concentration (ng/µL) ×10^− 9^] / [plasmid length× 660]). The plasmid concentration was adjusted to 10^6^ copies/µL with RNase-free water and stored at − 20 °C.

### Optimization of the pentaplex qRT-PCR reaction system and conditions

According to the manufacturer’s instructions, PCR reaction was performed with One Step PrimeScript™ RT-PCR Kit (Takara, Dalian, China). The primer and probe concentration, and Tm of the pentaplex qRT-PCR assay were optimized to set up the optimal reaction system and conditions for simultaneous detection of HBV, HCV, HEV, T. pallidum, and RNase P. In brief, PCR reactions were performed in 25 µL volumes, including 12.5 µL of 2X One Step RT-PCR Buffer III, 0.5 µL of Taq HS(5 U/µl),0.5 µL of PrimeScript RT Enzyme, 6.5 µL mixture of all primers and probes (0.2 µM of primers and probes for HBV, 0.1µM of primers and probes for HCV, T. pallidum, and RNase P, 0.4 µM of primers and probes for HEV), and 5 µL of nucleic acid template. PCR amplification was performed in the QuantStudio™ 5 Real-time PCR System(Applied Biosystems(ABI), Waltham, USA), and the conditions were: 42 °C for 5 min, a 10s denaturation step at 95 °C, followed by 40 cycles of 95 °C for 5s and 58 °C for 40s. The FAM, VIC, CY5, ROX, and TAMRA fluorescence signals intensity were recorded following each annealing step. Cycle threshold (Ct) values were generated with the QuantStudio Design & Analysis Software under automated threshold setting. The results were considered positive if the Ct value was within (≤) 38. Positive and negative controls were contained in each assay.

### Preparation of standard curves

To evaluate the linearity of the pentaplex qRT-PCR assay, serial tenfold dilutions of plasmid standards for HBV, HCV, HEV, T. pallidum, and RNase P ranging from 10^6^ to 10^1^ copies /µL were detected as templates and run in triplicate.

### The limit of detection of the pentaplex qRT-PCR

The nucleic acids of HBV, HCV, HEV, and T. pallidum, obtained from the State Key Laboratory for Diagnosis and Treatment of Infectious Diseases, China, were used to determine the limit of detection(LOD) of the pentaplex qRT-PCR. The nucleic acids were serially diluted into 100, 50, 10, 5, and 1 copies/µl and used as templates of the pentaplex qRT-PCR. For each template concentration, the assay was performed in 20 replicates. The results were analyzed using probit regression analysis. The LOD of the pentaplex qRT-PCR was considered as the lowest concentration level at which 95% of positive samples were detected.

### Specificity assessment of the pentaplex qRT-PCR

To assess potential cross-reactivity, the nucleic acids of HBV, HCV, HEV, T. pallidum, and related pathogens(HAV, EBV, HSV, CMV, HIV) were detected using the pentaplex qRT-PCR assay.

### Precision assessment of the pentaplex qRT-PCR

Plasmid standards of HBV, HCV, HEV, T. pallidum, and RNase P at six different concentrations (10^6^-10^1^ copies/µL) were used as templates of the pentaplex qRT-PCR to determine the precision of the assay. All templates were performed in triplicate for the intra-assay variability, while the coefficients of variation (CVs) in the inter-assay were determined on three different days during a week.

### Clinical performance of the pentaplex qRT-PCR assay

The clinical performance of the pentaplex qRT-PCR assay was assessed in 1200 blood samples from blood donors and 1200 blood samples from patients, respectively. 2400 blood samples were each in triplicate, one for the pentaplex qRT-PCR assay, one for the singleplex qPCR assay, and the other for serological testing. The singleplex qPCR and serological assays were performed by commercially available kits in accordance with the manufacturer’s protocols. The singleplex qPCR kits for detecting HBV DNA, HCV RNA, HEV RNA, and T. pallidum DNA were purchased from Shanghai Zhijiang Biotechnology Co., Ltd(Shanghai, China). The HBsAg, anti-HCV, IgM anti-HEV, and anti-T. pallidum enzyme-linked immunosorbent assay(ELISA) kits were obtained from Beijing Wantai Biological Pharmacy Enterprise Ltd(Beijing, China). These commercial test kits are approved by the National Medical Products Administration (NMPA) of China.

Nucleotide sequence assays were further performed in blood samples positive for either HBV DNA, HCV RNA, HEV RNA, or T. pallidum DNA to verify the results of the pentaplex qRT-PCR assay. Briefly, the amplified PCR products of blood samples positive for HBV, HCV, HEV, or T. pallidum were sequenced in single directions utilizing an ABI PRISM™ Big Dye™ Terminator Cycle Sequencing Kit (ABI, Foster City, CA, USA) through ABI 3730XL Genetic Analyzer (ABI). Then, the sequencing results were compared with the sequences published in GenBank through the genotyping tool (https://www.ncbi.nlm.nih.gov/projects/genotyping/formpage.cgi) for HBV and HCV genotypes identification. And HEV genotypes were identified with the HEVnet genotyping tool (https://www.rivm.nl/mpf/typingtool/hev/).

### Statistical analysis

Correlation coefficient (R^2^) values and amplification efficiencies(E) of standard curves were calculated with the QuantStudio Design & Analysis Software. The LOD of the pentaplex qRT-PCR was determined by probit regression analysis using SPSS 17.0 (SPSS Inc., Chicago, USA). Comparison of clinical performance between the pentaplex qRT-PCR and singleplex qPCR assay was evaluated by measuring sensitivity, specificity, and agreement using SPSS 17.0 (SPSS Inc., Chicago, USA).

## Results

### Standard curves of the pentaplex qRT-PCR assay

**Fig. 1:**
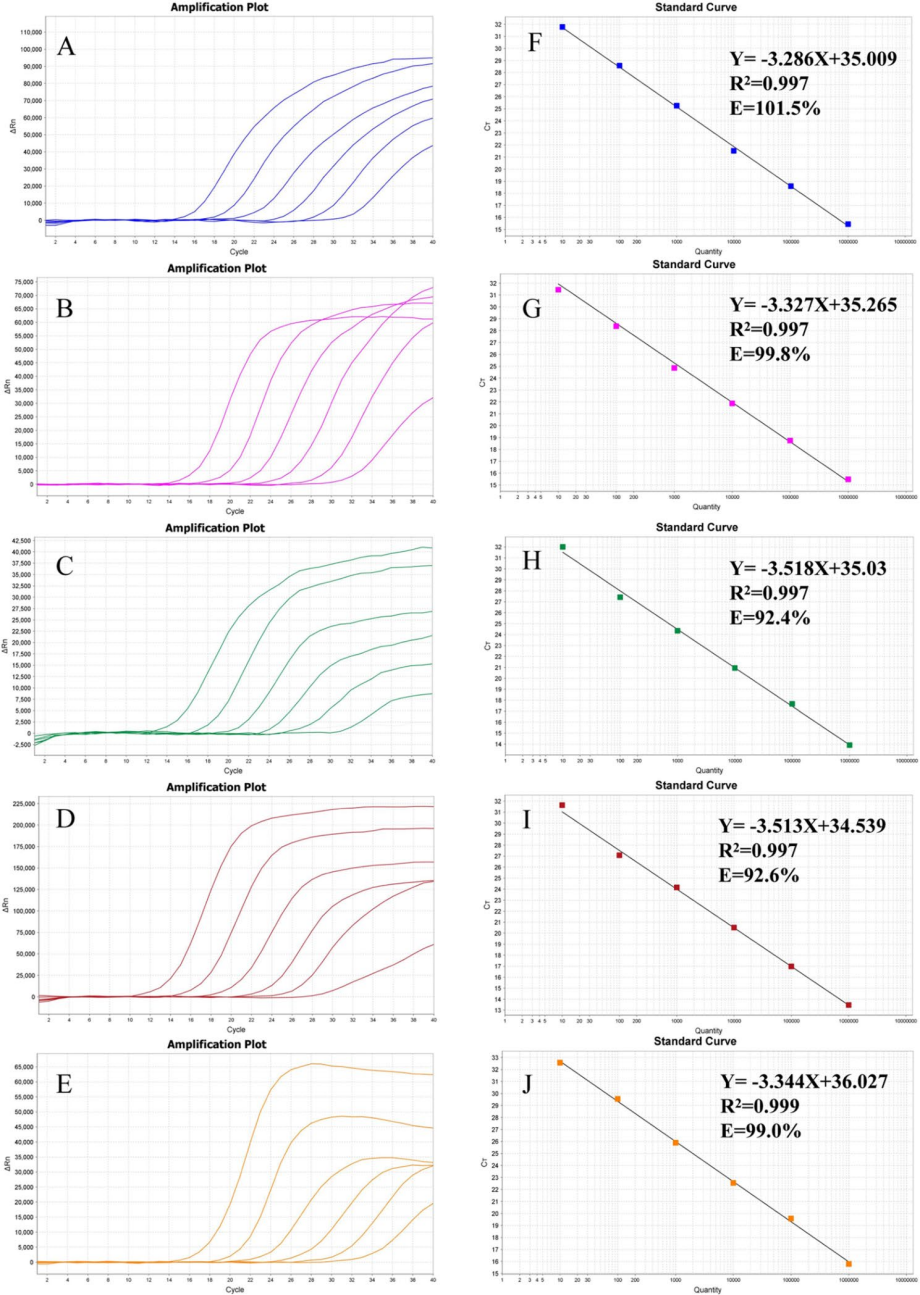
The amplification and standard curves of the pentaplex qRT-PCR assay. A, B, C, D, and E are the amplification curves of HBV, HCV, HEV, T. pallidum, and RNase P, respectively, using serial tenfold dilutions of plasmid standards ranging from 10^6^ to 10^1^ copies/µL, and F, G, H, I, and J are the standard curves of HBV, HCV, HEV, T. pallidum, and RNase P, respectively

The amplification and standard curves of the pentaplex qRT-PCR assay were obtained by serial tenfold dilutions of plasmid standards ranging from 10^6^ to 10^1^copies/µL as templates (Fig. 1). The correlation coefficient (R^2^) of standard curves of HBV, HCV, HEV, T. pallidum, and RNase P were 0.997,0.997,0.997,0.997, and 0.999, respectively, suggesting that the correlation between each quantified concentration group of standards is reliable. The amplification efficiencies(E) of targets were 101.5%,99.8%, 92.4%,92.6%, and 99.0%, respectively, regarded as satisfactory for the multiplex PCR method.

### The limit of detection of the pentaplex qRT-PCR

The LOD of the pentaplex qRT-PCR was determined by testing serial dilutions of the nucleic acids of HBV, HCV, HEV, and T. pallidum. The hit rates of five different template concentrations(100, 50, 10, 5, and 1 copies/µL) in 20 replicates were measured and listed in Table 2. The levels of 95% LOD were obtained using the probit regression analysis: 7.11 copies/µL for HBV, 7.65 copies/µL for HCV, 8.45 copies/µL for HEV, and 9.06 copies/µL for T. pallidum.

**Table 2:** The limit of detection of the pentaplex qRT-PCR

Pathogen	Concentration (copies/µL)	Reactions	Positive	Hit rate(%)	95%LOD (copies/µL)
HBV	100	20	20	100	7.11
	50	20	20	100	
	10	20	20	100	
	5	20	17	85	
	1	20	14	70	
HCV	100	20	20	100	7.65
	50	20	20	100	
	10	20	20	100	
	5	20	16	80	
	1	20	11	55	
HEV	100	20	20	100	8.45
	50	20	20	100	
	10	20	20	100	
	5	20	14	70	
	1	20	8	40	
T. pallidum	100	20	20	100	9.06
	50	20	20	100	
	10	20	20	100	
	5	20	10	50	
	1	20	3	15	

### The analytical specificity of the pentaplex qRT-PCR

**Fig. 2:**
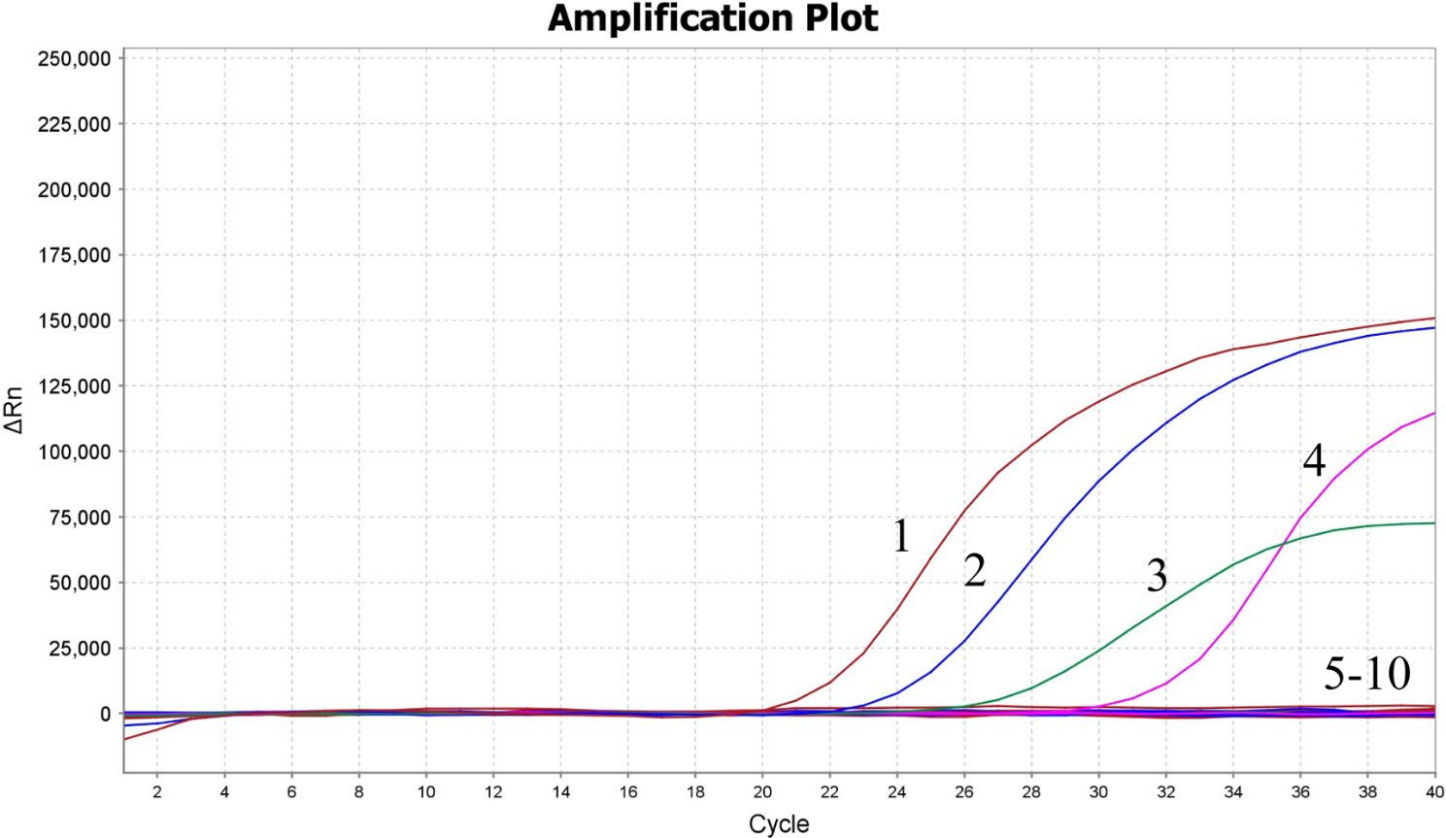
The analytical specificity of the pentaplex qRT-PCR assay. Only HBV, HCV, HEV, and T. pallidum had amplification curves. HAV, EBV, HSV, CMV, HIV, and negative controls had no fluorescence signal.1, T. pallidum; 2, HBV; 3, HEV; 4, HCV; 5–9, HAV, EBV, HSV, CMV, and HIV; 10, negative control

The DNA or RNA of HBV, HCV, HEV, T. pallidum, HAV, EBV, HSV, CMV, and HIV were used as templates of the pentaplex qRT-PCR to evaluate the specificity of the assay. The results illustrated that only HBV, HCV, HEV, and T. pallidum had specific amplification curves, while HAV, EBV, HSV, CMV, and HIV did not demonstrate any positive signals and amplification curves, indicating high specificity of the pentaplex qRT-PCR (Fig. 2).

### The precision of the pentaplex qRT-PCR

The CVs of intra-assay and inter-assay were tested to assess the precision of the pentaplex qRT-PCR assay. The results showed that both intra-assay and inter-assay CVs were less than 2% (Table 3), indicating good repeatability (intra-assay) and reproducibility (inter-assay) of the assay.

**Table 3:** Intra-assay and inter-assay coefficients of variation for the pentaplex qRT-PCR

Target(copies/µL)	Intra-assay CV(%)					Inter-assay CV(%)				
	HBV	HCV	HEV	T.pallidum	RNase P	HBV	HCV	HEV	T.pallidum	RNase P
10^6^	1.69	0.68	1.60	1.13	1.74	1.66	1.05	1.72	1.93	2.00
10^5^	1.19	0.57	1.17	0.94	1.35	1.63	0.68	1.84	1.57	1.48
10^4^	1.08	0.42	0.93	0.78	0.60	1.67	0.80	1.52	1.23	1.10
10^3^	0.29	0.18	1.47	0.50	0.85	1.58	1.20	1.66	0.89	1.17
10^2^	0.75	0.17	0.86	0.73	0.59	0.83	0.69	1.01	1.13	0.62
10^1^	0.44	0.33	1.30	0.64	1.61	0.66	0.98	1.74	1.81	1.61

### Consistency between the pentaplex qRT-PCR assay and commercial singleplex qPCR kits in blood samples

The clinical performance of the pentaplex qRT-PCR and commercial singleplex qPCR assay were compared in 2400 blood samples from blood donors and patients. RNase P was positive in all blood samples. As shown in Table 4, in 1200 blood donors, the pentaplex qRT-PCR assay identified 13(1.08%) positive for HBV DNA, 3(0.25%) positive for HCV RNA, and 1(0.08%) positive for HEV RNA. The pentaplex qRT-PCR assay detected 56(4.67%) positive for HBV DNA and 9(0.75%) positive for HCV RNA in 1200 clinical samples, with no detectable HEV or T. pallidum. Moreover, one clinical sample was identified as HBV/HCV double positive. The results of the pentaplex qRT-PCR assay were in agreement with those obtained from the same samples with commercial singleplex qPCR assay. These positive samples identified by the pentaplex qRT-PCR assay were confirmed as true positives by sequencing. Therefore, the pentaplex qRT-PCR assay for detecting HBV, HCV, HEV, and T. pallidum in blood presented 100% clinical sensitivity, specificity, and consistency, when compared to commercial singleplex qPCR assay.

Sequencing results showed that 21 (30.43%) of all HBV positive samples were HBV genotype B, 48(69.57%) were HBV genotype C, all HCV positive samples were genotype 1(12,100%), and one HEV positive sample was genotype 4(1,100%).

**Table 4:** Consistency of the pentaplex qRT-PCR and singleplex qPCR for detecting blood samples

	No. (%) positive numbers in blood donors				No. (%) positive numbers in clinical samples			
Subject	HBV	HCV	HEV	T. pallidum	HBV	HCV	HEV	T. pallidum
Pentaplex qRT-PCR	13(1.08)	3(0.25)	1(0.08)	0(0)	56(4.67)	9(0.75)	0(0)	0(0)
Singleplex qPCR	13(1.08)	3(0.25)	1(0.08)	0(0)	56(4.67)	9(0.75)	0(0)	0(0)
Sensitivity (% )	100%	100%	100%	100%	100%	100%	100%	100%
Specificity (% )	100%	100%	100%	100%	100%	100%	100%	100%
Agreement (% )	100%	100%	100%	100%	100%	100%	100%	100%

### Comparison of the pentaplex qRT-PCR and serological assays in blood samples

As shown in Table 5, in 1200 blood donors, a positive serological assay for HBsAg was present in 13 (1.08%), anti-HCV was positive in 4 (0.33%), IgM anti-HEV was positive in 19 (1.58%), and anti-T. pallidum was positive in 1 (0.08%). The serological assays showed the presence of HBsAg in 57 (4.75%), the presence of anti-HCV in 11 (0.92%), the presence of IgM anti-HEV in 10 (0.83%), and the presence of anti-T. pallidum in 5 (0.42%) of the 1200 clinical samples.

**Fig. 3:**
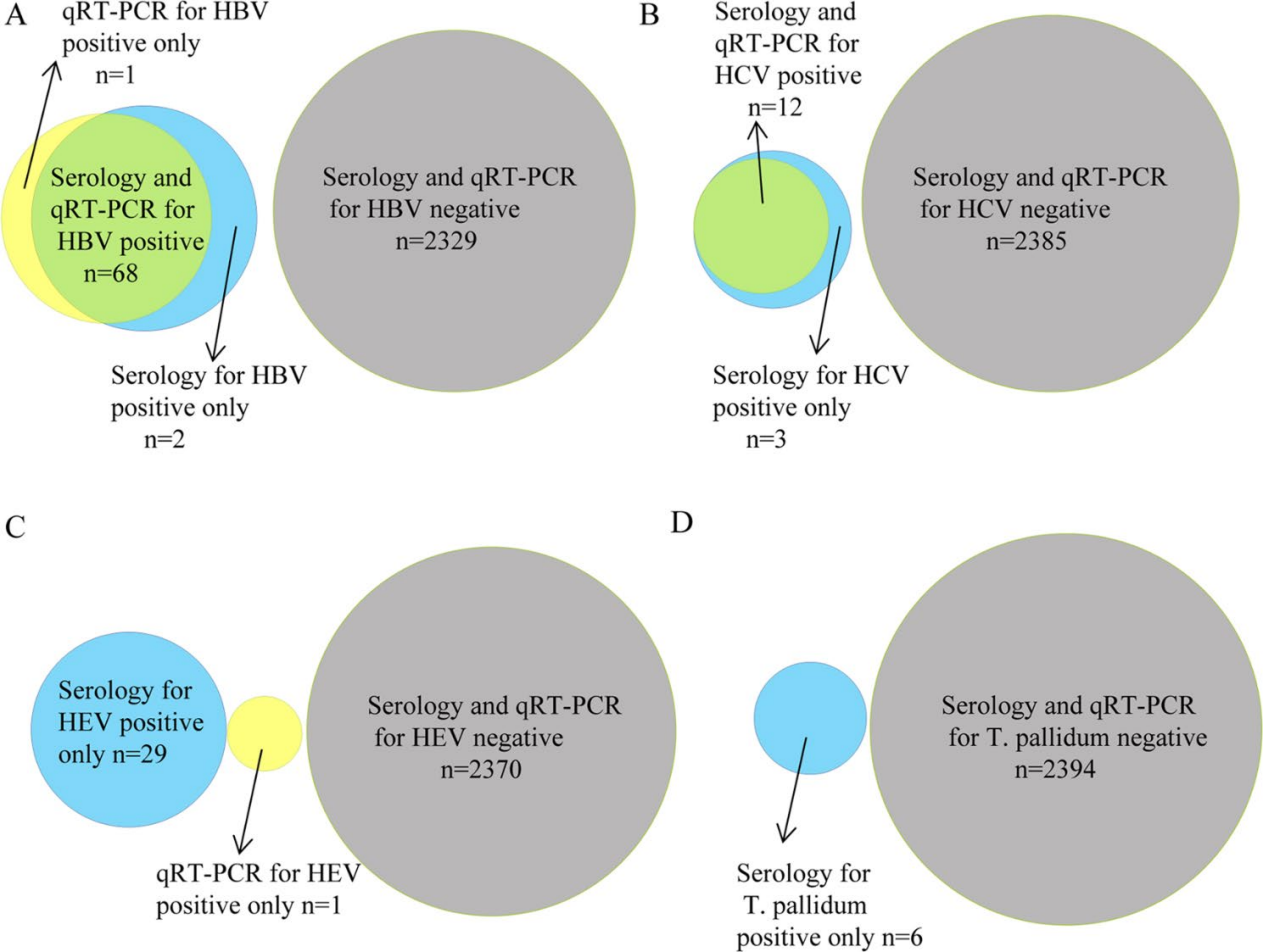
Venn diagram of the results of serology versus pentaplex qRT-PCR assays. The serology and pentaplex qRT-PCR assays were performed to detect HBV(A), HCV(B), HEV (C), and T. pallidum(D) in 2400 blood samples. Number of samples positive by serology only (blue), positive by pentaplex qRT-PCR only(yellow), or positive by both serology and pentaplex qRT-PCR (green). Number of samples negative by both serology and pentaplex qRT-PCR(grey)

Nevertheless, there were several different results between the serological and pentaplex qRT-PCR assays detection of HBV, HCV, HEV, and T. pallidum in 2400 blood samples (Fig. 3) .12(0.50%) blood donors and 56(2.33%) clinical samples were positive for both the HBsAg and HBV DNA.1(0.04%) blood donor and 1(0.04%) clinical sample were HBsAg-positive but HBV DNA–negative, and 1(0.04%) blood donor was HBV DNA-positive but HBsAg-negative.The number of samples with positive anti-HCV and HCV RNA was 12(0.50%), including 3(0.13%) blood donors and 9(0.38%) clinical samples.1(0.04%) blood donor and 2(0.08%) clinical samples with positive anti-HCV were negative for HCV RNA. 19(0.79%) blood donors and 10(0.42%) clinical samples of IgM anti-HEV positive were all negative for HEV RNA, but 1(0.04%) blood donor of HEV RNA positive was tested negative for IgM anti-HEV. 1(0.04%) blood donor and 5(0.21%) clinical samples of anti-T. pallidum positive were all negative for T. pallidum DNA.

**Table 5:** Serological assays for blood donors and clinical samples

	Blood donors	Clinical samples	Total
HBsAg positive(No. (%) )	13(1.08)	57(4.75)	70(2.92)
Anti-HCV positive(No. (%) )	4(0.33)	11(0.92)	15(0.63)
IgM anti-HEV positive(No. (%) )	19(1.58)	10(0.83)	29(1.21)
Anti-T. pallidum positive(No. (%) )	1(0.08)	5(0.42)	6(0.25)

## Discussion

In the present study, we have developed a pentaplex qRT-PCR assay for the simultaneous detection of HBV, HCV, HEV, T. pallidum, and RNase P. The RNase P was applied as an internal control to assess possible inhibition in PCR assay and prevent false negative results from test failure. As far as we know, this is the first report on the simultaneous detection of HBV, HCV, HEV, and T. pallidum in a single closed test tube.

Primers and probes in this work were designed towards the S region of HBV, 5’ UTR of HCV, ORF2 region of HEV, TPP47 region of T. pallidum, and RPP30 region of RNase P, respectively, which have been identified as the ideal areas for the development of PCR assays, because of their low profile of mutations between different genotypes/subtypes. These highly conservative genes are also the commonly used targets for PCR in previous reports [24, 26, 28]. The 95% LOD were 7.11 copies/µL for HBV, 7.65 copies/µL for HCV, 8.45 copies/µL for HEV, and 9.06 copies/µL for T. pallidum, which were similar to the results of other studies [27, 29]. In addition, this assay has shown high analytical specificity (Fig. 2) and precision (Table 3).

The clinical performance of the developed pentaplex qRT-PCR assay was evaluated in 2400 blood samples. Compared to commercial singleplex qPCR assay, the pentaplex qRT-PCR for detecting HBV, HCV, HEV, and T. pallidum presented 100% clinical sensitivity, specificity, and consistency. The developed pentaplex qRT-PCR was more sensitive than multiplex RT-PCR for simultaneous detection of HBV, HCV, and HEV developed by Garg et al., clinical sensitivity of which were 88.9 ± 4% for HBV, 87.59 ± 8% for HCV, and 73.9 ± 8% for HEV in comparison to singleplex qPCR, respectively. The clinical specificity of the pentaplex qRT-PCR was identical to multiplex RT-PCR developed by Garg et al. [30].

Average detection time for 96 samples in one run was 65 min for the pentaplex qRT-PCR assay. The time and cost for one sample were about one quarters of that of singleplex qPCR assays for detecting HBV, HCV, HEV, and T. pallidum in separate individual reaction. One sample was identified as HBV DNA/HCV RNA double positive by the pentaplex qRT-PCR assay only in a single tube. The study indicates that the novel assay effectively identifies HBV, HCV, HEV, and T. pallidum independently in one run, which provides technical support for the rapid, economical, and accurate diagnosis of individual and mixed pathogens infections.

Genotype analysis results showed that HBV positive samples were genotype B(21,30.43%) and C(48,69.57%), all HCV positive samples were genotype 1(12,100%), and HEV positive sample was genotype 4(1,100%), which were consistent with genotype distribution reported for China. The HBV B and C genotypes, HCV 1b and 1a genotypes, and HEV 1 and 4 genotypes are most prevalent in China, respectively [31–33].

In the present study, 2(0.08%) samples confirmed HBsAg-positive but HBV DNA-negative owing to low viral load. One blood donor was identified as HBV DNA-positive but HBsAg-negative, presumably pre-seroconversion window period or occult infection, suggesting that the absence of HBsAg does not completely exclude the existence of the virus. The inconsistent results emphasized the importance of HBV DNA detection in the HBsAg-negative infection window period. Several reports have confirmed the ability of NAT to detect occult HBV infection and inhibit transfusion-transmitted HBV [34, 35].

Anti-HCV detection does not distinguish between those recovered from previous infection and those with current infection, while HCV RNA testing is acknowledged as the gold standard for confirming active HCV infection [36]. 3(0.13%) anti-HCV positive samples were tested negative for HCV RNA. The discrepant results could be attributed to either past infection, levels of RNA concentrations in samples below the LOD of PCR assay, or false positives. HyperIgemia and rheumatoid factor interference may lead to serological false positive results [37].

In this study, 29(1.21%) samples with IgM anti-HEV positive were all negative for HEV RNA, which could be due to low viral load, IgM persistence of unknown causes for several years after HEV primary infection, or false positives resulting from cross-reaction with other viruses and rheumatoid factor interference [27]. Previous studies have shown that only a minority donors of anti-HEV IgM positive were detected positive for HEV RNA [38, 39]. In addition, we found that one HEV RNA-positive blood donor was tested negative for IgM anti-HEV, which indicated that HEV RNA testing might help improve screening efficiency and blood safety. 72.15%(57/79) of viremic donors were seronegative for HEV reported by Tedder et al. [40]. Recently, a comprehensive meta-analysis showed that HEV RNA screening of blood donors, especially in HEV endemic areas, might reduce the potential risk of transfusion-transmitted HEV [33]. Although our results show a risk of transfusion-transmitted HEV infection, extensive research is still needed to demonstrate the necessity of HEV screening for blood donors in China.

Anti-T. pallidum persists in the body after the successful cure of syphilis. Serological testing may produce false positive results under autoimmune disease and virus infection [41]. This study showed 6 (0.25%) samples with anti-T. pallidum positive were negative for T. pallidum DNA, which may ascribe to successful treatment, biological false positives, or low T. pallidum DNA loading in the secondary and latent stages of syphilis. Vrbova et al. proposed that PCR detection rates were low in the secondary and undetermined stages of syphilis [15].

This experiment has some limitations. First, although the primers and probes in this study were designed towards highly conserved regions of each target, the possibility of gene mutations, deletions, and genetic recombination over time results in off-target primers and probes, which further leads to false negative results. Second, the sample size was relatively small. It is also necessary to verify the pentaplex qRT-PCR assay in more samples.

## Conclusions

In conclusion, we established a one-step pentaplex qRT-PCR assay for simultaneously, sensitively, specifically, and reproducibly detecting HBV, HCV, HEV, and T. pallidum, and also detecting a human housekeeping gene (RNase P) as an internal control to ensure test validity. The developed pentaplex qRT-PCR assay has the advantages of high sensitivity and specificity, high throughput, less time and labor consumption, and cost-effectiveness. It might be of great potential for large-scale screening of blood donors, clinical diagnosis, and epidemiological studies of pathogens.

## Data Availability

The datasets used and/or analysed during the current study available from the corresponding author on reasonable request. The datasets generated and/or analysed during the current study are available in the GenBank repository, GenBank accession numbers: OQ660350-OQ660431.

## Abbreviations

HBV: Hepatitis B virus
HCV: Hepatitis C virus
HEV: Hepatitis E virus
T. pallidum: Treponema pallidum
qRT-PCR: real-time reverse transcription PCR
LOD: limit of detection
WHO: World Health Organization
NAT: nucleic acid testing
TTIs: transfusion-transmitted infections
HBsAg: Hepatis B surface antigen
anti-HCV: Anti-Hepatitis C virus
CVs: coefficients of variation
HAV: hepatitis A virus
EBV: Epstein-Barr virus
HSV: herpes simplex virus
CMV: cytomegalovirus
HIV: human immunodeficiency virus
ELISA: enzyme-linked immunosorbent assay
VIC: 2’-chloro-7’-phenyl-1,4-dichloro-6-carboxyfluorescein
Cy5: Cyanine5
TAMRA: 6-carboxy-tetramethyl-rhodamine
FAM: 6-carboxyfluorescein
BHQ1: 8-Bromo-7-hydroxyquinoline1
BHQ2: 8-Bromo-7-hydroxyquinoline2
BHQ3: 8-Bromo-7-hydroxyquinoline3
S: surface
5’ UTR: 5’ untranslated region
ORF2: open reading frame-2
TPP47: T. pallidum 47 kDa lipoprotein
RPP30: ribonuclease P protein subunit p30
ABI: Applied Biosystems
NMPA: National Medical Products Administration
